# Predictors of resilience in deaf and hard of hearing adolescents and adolescents with developmental language disorders

**DOI:** 10.3389/fpsyg.2026.1773481

**Published:** 2026-06-26

**Authors:** Len C. Martijn, Daan Hermans, Harry E. T. Knoors, Constance T. W. M. Vissers

**Affiliations:** 1 Stichting VierTaal, VierTaal College Amsterdam, Amsterdam, Noord-Holland, Netherlands; 2 Behavioural Science Institute, Radboud University, Nijmegen, Gelderland, Netherlands; 3 Koninklijke Kentalis, Utecht, Netherlands

**Keywords:** deaf, developmental language disorders, executive functions, hard of hearing, resilience, schemas, support, theory of mind

## Abstract

**Background:**

Many adolescents with communication problems (CP), in this case, deaf and hard of hearing (DHH) adolescents and those with developmental language disorders (DLD), tend to experience high levels of stress in their daily lives. Resilience is seen as a protective mechanism that supports adaptive functioning and helps navigate situations involving increased stress; however, little appears to be known about this group’s resilience and the factors that contribute to it.

**Objective:**

This study assesses resilience and the predictive value of four factors contributing to resilience, dysfunctional schemas, theory of mind (ToM), executive functions (EFs), and social support, in adolescents with CP (DHH, DLD).**Participants and setting:** A total of 127 adolescents with CP (32 DHH adolescents and 95 with DLD) in special secondary education were assessed. To establish a normative baseline for resilience, a reference group of 86 adolescents without CP in mainstream secondary education was recruited for comparative analysis.

**Methods:**

Two-sided *t*-tests and *z*-tests were used to compare all groups on resilience, and two-sided *t*-tests were performed to compare DHH adolescents with adolescents with DLD on dysfunctional schemas, ToM, EFs, and social support. Two multiple linear regression analyses, one with and one without controlling for adolescents’ type of communication problems, were conducted to examine the contributions of dysfunctional schemas, ToM, EFs, and social support to resilience.

**Results:**

Adolescents with CP reported significantly lower resilience than the reference group (*p* < 0.001). No differences were found in the reported resilience, dysfunctional schemas, ToM, EFs, and social support between DHH adolescents and adolescents with DLD (*p* > 0.1). A multiple linear regression analysis revealed dysfunctional schemas as the only significant predictor of resilience (*p* < 0.001), with a standardized beta coefficient of −0.57 indicating a strong negative relationship.

**Conclusion:**

Adolescents with CP appear to be a quite vulnerable group of youth with low resilience to protect them from the negative effects of stress. Their dysfunctional schemas seem to serve as a predictive factor of their resilience.

## 1 Introduction

Adolescents with communication problems (CP), such as deaf and hard of hearing adolescents (DHH) and adolescents with developmental language disorders (DLD), appear to be vulnerable to experiencing stress. While they may experience stress related to their communication problems (Burnley et al., 2023; Hall et al., 2023), they may also be subject to additional stress as adolescents with CP in special education appear more susceptible to adverse childhood experiences (ACEs) than adolescents without CP in mainstream education (Martijn et al., 2025). Resilience is viewed as a protective mechanism that enables adaptive functioning and navigation through contexts of heightened stress, adversity, or trauma (Masten et al., 2021; Millican and Middleton, 2020; Shonkoff et al., 2021). Rather than an inherent trait, resilience refers to the dynamic ability to maintain reasonable, stable mental health during or after a traumatic event or cumulative adversity. It also involves having a strong capacity to adapt to life’s challenges (Boyce et al., 2021; Center of the Developing Child, 2025; Masten, 2018, 2016; Shafi et al., 2020). Because adolescents with CP seem more vulnerable to experiencing stress, this study aims to gain more insight into their resilience and the factors that positively contribute by identifying protective factors that can be leveraged to foster resilience.

Studies into resilience in children, adolescents, or adults with CP (DHH and DLD) are scarce. Only four studies were identified involving DHH participants; two of which compared resilience between DHH and typical-hearing participants. Butler et al. (2018) evaluated resilience as an outcome factor and found no differences between 30 DHH adolescents, visually impaired adolescents, and typically developing adolescents. Bizuneh (2022) examined resilience as an outcome variable in 80 DHH adolescents and found that they had significantly lower resilience than a hearing control group. Eichengreen et al. (2022) focused on adults’ retrospective perspectives on resilience during childhood and adolescence. They conducted semi-structured interviews with 23 DHH young adults regarding familial, social, and personal aspects of coping with hearing loss. The results suggested that positive relationships, self-attitudes, and coping strategies fostered resilience. A study by Ashori and Aghaziarati (2022) examined 110 DHH preschool children, comparing their empathy and behavior problems with their resilience. A significant positive relationship was found between empathy and resilience, while a significant negative relationship was observed between behavior problems and resilience. Studies specifically assessing resilience in populations with DLD appear even sparser. One study evaluated the wellbeing and resilience of 11 children through a semi-structured interview; the authors noted that communication impairment, interpersonal difficulties, and concerns about academic achievement could be potential risk factors for wellbeing and suggested that hope, agency, and positive relationships could serve as protective factors for resilience (Lyons and Roulstone, 2018).

Resilience stems from our neurobiological stress regulation and response system, which develops during gestation and continues to develop after birth, especially during the early years of brain plasticity when children develop skills for coping and adapting to different experiences (Malhi et al., 2019; Masten, 2018; Mesman et al., 2021). As a dynamic construct, research on resilience development has identified various internal and external factors that contribute to resilience (Troy et al., 2023). Building on this research, the present study focused on four factors that are frequently described as contributing to resilience: three internal factors: schemas, theory of mind, and executive functions, and one external factor: social/emotional support (Bak et al., 2015; Parsons et al., 2016; Pinto et al., 2021; Racine et al., 2022; Riepenhausen et al., 2022; Yao and Hsieh, 2019).

Schemas, or core beliefs, are fundamental cognitive frameworks through which individuals interpret and evaluate how they perceive themselves, others, and the world around them. Schemas are shaped by emotions, memories, beliefs, and sensations, all of which profoundly affect the individual’s coping responses (thoughts, feelings, and behavior) (Korrelboom and ten Broeke, 2004; Young et al., 2003). Schemas can be functional or dysfunctional. When facing life’s challenges, functional or healthy schemas can help a person navigate through psychological distress (Joosten et al., 2024; Keyfitz et al., 2013; Young et al., 2003). Positive beliefs, such as “I have confidence in myself” or “I am worthy of love,” can help maintain self-esteem and self-efficacy. Beliefs like “I belong” or “people are generally trustworthy” may encourage seeking social support in crises, and competence beliefs like “I can deal with problems” may support adaptation to challenges. In contrast, dysfunctional schemas may increase vulnerability to psychological distress when encountering life’s difficulties. Negative beliefs like “I am not worthy of love,” “people cannot be trusted,” or “I am a failure” can undermine self-esteem, hinder a person’s ability to adequately adapt to challenges, and may trigger maladaptive coping strategies, such as avoiding dealing with adversities (Young et al., 2003). Research indicates that healthy/functional schemas are positively associated with resilience (Joosten et al., 2024; Keyfitz et al., 2013), whereas dysfunctional schemas are negatively associated with resilience (Friedmann et al., 2016; Tariq et al., 2021; Young et al., 2003). For example, someone with a dysfunctional schema of unrelenting standards might believe they must perform at an exceptionally high level on academic tasks. Receiving a failing grade can be perceived as a personal disappointment and failure, which may lead to a lasting sense of inadequacy. Regarding schemas among DHH youth or youth with DLD, to the best of our knowledge, no studies have been conducted.

Theory of Mind (ToM) refers to the cognitive ability to attribute mental states to oneself and others, such as thoughts, beliefs, emotions, desires, and intentions (Rakoczy, 2022; Wade et al., 2018). This ability supports understanding that others may have perspectives different from one’s own. The relationship between ToM and resilience can be understood within the broader context of social and emotional competence. A well-developed ToM improves an individual’s ability to navigate social interactions, skills that are essential for adaptive coping and, by extension, resilience (Rakoczy, 2022; Ruffman, 2023). For example, an adolescent with low self-worth (schema) who is left out of a social event may perceive this as proof of being less worthy. They might believe peers intentionally aimed to hurt them and verbally lash out, which can lead to further exclusion and reinforce their low self-worth. With proficient ToM, the adolescent might still feel excluded and interpret it as personal; however, proficient ToM abilities can help them respond appropriately in social situations, even when their personal beliefs and feelings differ. Regarding ToM in children and adolescents with CP, a systematic review of 97 papers (Martín et al., 2025) examined and compared different subgroups of DHH participants alongside typically hearing counterparts in the age range of 1–96 years. The results revealed that typically hearing participants generally performed better than their DHH counterparts on ToM assessments. However, significant variability was observed among DHH participants, influenced by factors such as the quality of early interactions and the timing of early exposure to both signed and spoken language. In children with DLD, a systematic review and meta-analysis of 17 studies involving 745 children aged 4–12 years found significantly lower ToM performance compared to their age-matched, typically developing peers (Nilsson and de López, 2016). The analysis revealed that neither age nor gender explained this performance gap. One study was identified that compared adolescents with CP. Smit et al. (2022) compared 14 DHH adolescents and 13 adolescents with DLD in special secondary vocational education, along with a control group of typically developing peers. Although DHH adolescents showed lower ToM abilities than the control group, their scores were as consistent as those of the control group, whereas adolescents with DLD showed no differences in ToM abilities compared to the control group, but their scores were more inconsistent than those of the control group.

Executive functions (EFs) include a set of cognitive processes essential for goal-directed behavior, and EFs facilitate the regulation of negative thoughts to adopt a more positive outlook when necessary. The theoretical framework linking EFs to resilience lies in processes that facilitate the retrieval of intentions and flexibly overriding automated responses in favor of new, goal-directed behavior, thereby enabling adaptation to unfamiliar or challenging circumstances (Diamond, 2013; Gilbert and Burgess, 2008). Research on the association between EFs and resilience suggests that resilience is not associated with a single executive function; rather, multiple EFs are indicated to contribute to resilience. For instance, cognitive functions such as working memory and mental flexibility contribute to adaptive functioning, while behavioral functions like inhibitory control and emotion regulation help regulate negative thoughts and inhibit negative behavior (Mecha et al., 2024; Wu et al., 2021). The different EFs are both interrelated and partially independent constructs (Miyake et al., 2000). By facilitating flexible and effective problem-solving, these cognitive and behavioral functions seem to serve as mechanisms for mitigating negative outcomes, an essential component of resilience (Kalisch et al., 2015, Kalisch et al., 2019; Mecha et al., 2024; Obradović, 2016; Wu et al., 2021). An example: During a tense moment in a soccer match, when a hard tackle results in an injury and a goal for the opposing team, impulse control and emotion regulation are crucial for an adolescent. This allows them to avoid retaliating with a tackle or swearing at the referee, risking a red card, thus learning to cope with setbacks. Morgan and Dye (2020) reviewed studies on the EFs of DHH children in relation to their language development. The findings indicate that the development of EFs is influenced by early access to fluent language and high-quality communicative interactions. DHH children born to deaf parents who acquire natural sign language early on often develop typical EF skills similar to their hearing peers. However, most DHH children are born to hearing parents and appear to be at a higher risk for delayed EFs development. Although DHH children born to hearing parents usually perform worse on EFs tasks compared to their typically developing peers, there is considerable variation in study results across different EFs tasks. In children with DLD, a systematic review and meta-analysis by Niu et al. (2024) involving 40 studies with children aged 3.4–11.5 years showed that they perform significantly poorer on all examined verbal working memory tasks compared to their typically developing peers. On other EFs tasks, inhibitory control and cognitive flexibility, the results were less conclusive, as differences were observed between studies.

In addition to these internal factors (dysfunctional schemas, ToM, and EFs), the present study also focused on a commonly cited external factor that contributes to resilience: social/emotional support. Meta-analyses conducted over the past three decades have consistently demonstrated a protective effect of social/emotional support in buffering against increasing stress (Cohen and Wills, 1985; Masten, 2018; Vila, 2021; Yule et al., 2019; Zalta et al., 2021). This research indicates that in (early) childhood, resilience is fostered through supportive, responsive, and protective relationships with attachment figures, such as parents and caretakers (Masten, 2018; Center of the Developing Child, 2025). During adolescence, it is believed that the moderating effect of perceived social/emotional support on stress regulation and resilience shifts from attachment figures to friends and important others, such as teachers (Vila, 2021; Zalta et al., 2021). An example: Open communication with a parent, teacher, or trusted friend can help an adolescent reframe setbacks as opportunities for growth rather than personal failures. Such relationships build a secure emotional base, enabling adolescents to navigate challenges with greater confidence. In adolescents with CP only two studies were identified on social/emotional support. A survey by Piso et al. (2009) compared 12 DHH adolescents aged 13–19 years with a reference group of typically developing peers who were all attending mainstream education. The DHH adolescents reported no differences compared to their typically developing peers in the level of perceived support from key people in their social network. In adolescents with DLD a study by Forrest et al. (2021) examined perceived social support in friendships between 26 adolescents with DLD and 27 age- and gender-matched controls. The researchers found that these groups reported similar levels of perceived social support from friends.

When considering the studies regarding schemas, ToM, EFs, and social support in children and adolescents with CP, notable disparities emerge in the extent to which these factors have been examined in this group.

The limited research on resilience in adolescents with CP necessitates that the current study starts by assessing resilience within this group. Drawing upon data from the aforementioned studies concerning factors that contribute to resilience, ToM, and EFs, it can be hypothesized that resilience is likely to be compromised in many adolescents with CP, given the reported difficulties in these key areas. Therefore, it is also hypothesized that these factors predict resilience. Since only two studies on social/emotional support in populations with CP were identified, and none were found regarding dysfunctional schemas in this group, an exploratory approach is used to assess adolescents’ dysfunctional schemas and perceived social/emotional support in addition to its predictive value for resilience.

Because the relationship between the four factors (dysfunctional schemas, ToM, EFs, and social/emotional support) and resilience in this group appears unexamined, this study is designed to assess the extent to which these factors contribute to resilience. This methodology aims to determine whether variation in resilience is equally explained by variation in dysfunctional schemas, ToM, EFs, or social/emotional support, or if one specific factor is a stronger predictor. Since it is hypothesized that adolescents with CP may have compromised resilience, findings on the predictive value of these factors may help inform interventions aimed at improving resilience in these youths. Furthermore, this approach will enable a direct comparison between DHH adolescents and adolescents with DLD concerning resilience and the four predictive factors. While shared early communication problems suggest commonality across both groups, it may also be posited that group-specific factors, such as the type of communication problems related to being deaf, hard of hearing, or having DLD, could account for differences. Differences in the implementation of early interventions (often earlier for DHH children, Boerma et al., 2023; Knoors and Marschark, 2013) or in growing up with a different language modality than the people around them (most DHH children have hearing parents and teachers, Knoors and Marschark, 2013) may influence resilience development.

This study’s assessment of resilience and the four predictive factors focuses on adolescents with CP in special secondary education. In the Netherlands, students with CP qualify for special education when their educational needs cannot be met in mainstream education with support from a peripatetic teacher. Approximately 50% of all students with CP are educated in special settings tailored to their communication needs.

In summary, this study aims to answer the following questions: How resilient are adolescents with CP (DHH adolescents and adolescents with DLD)? To what extent do internal and external factors (dysfunctional schemas, ToM, EFs, social/emotional support) contribute to resilience in adolescents with CP? Additionally, the current study explores whether the type of communication problems in adolescents with CP leads to differences in the relationships between internal and external factors and resilience.

## 2 Materials and methods

### 2.1 Study design

A two-group comparison design was used to compare DHH adolescents and adolescents with DLD on resilience. To establish a normative baseline concerning resilience, a reference group (RG) of adolescents without CP in mainstream secondary education was recruited for a comparative analysis. Regarding the predictors of resilience (dysfunctional schemas, ToM, EFs, and social support), a two-group comparison design was also employed to compare the DHH participants with those with DLD. The study included questionnaires addressing resilience, schemas, ToM, EFs, and social networks.

### 2.2 Participants

In special secondary education, participants with CP were recruited; DHH adolescents (*n* = 32, *M* age = 14.5 years, *SD* = 1.87, range: 12–17) and adolescents with DLD (*n* = 95, *M* age = 14.5 years, *SD* = 1.56, range: 12–17). DHH participants demonstrated a diverse range of auditory profiles. Clinical classifications included mild (26–40 dB HL; *n* = 4), moderate (41–70 dB HL; *n* = 13), severe (71–90 dB HL; *n* = 6), and profound (>91 dB HL; *n* = 9) hearing loss. Regarding auditory amplification, configurations included bilateral hearing aids (*n* = 17), unilateral hearing aids (*n* = 4), bilateral cochlear implants (*n* = 3), and unilateral cochlear implants (*n* = 2). Additionally, six participants used a bimodal fitting (a hearing aid and a cochlear implant). The reference group was recruited in mainstream secondary education; adolescents without CP (*n* = 86, *M* age = 14.2 years, *SD* = 1.66, range: 12–17) (see demographic details in Table 1).

**TABLE 1:** Demographic characteristics of participants.

Participants	CP	RG			DHH	DLD		
Demographics	%	%	*z*	Two-sided *p*	%	%	*z*	Two-sided *p*
Gender								
Male	62.99	62.79	0.030	0.976	65.63	62.11	0.357	0.721
Female	28.35	33.72	−0.836	0.403	25.00	29.47	−0.486	0.627
Neutral/no answer	8.66	3.49	1.495	0.135	9.38	8.42	0.166	0.868
Education								
Vocational	33.86	23.26	1.664	0.096	31.25	34.74	−0.360	0.718
Vocational (basic-advanced)	52.76	10.47	6.321	<0.001**	40.63	56.84	−1.589	0.112
General vocational	9.45	25.58	−3.154	0.002*	18.75	6.32	2.080	0.038*
Senior general	3.94	40.70	−6.740	<0.001**	9.38	2.11	1.829	0.067

*N* = 213. Reference group, RG *n* = 86. Adolescents with CP, CP *n* = 127. DHH *n* = 32, DLD *n* = 95.

*p < 0.05.

**p < 0.001.

### 2.3 Procedure

All special secondary schools in the Netherlands that cater to students with CP were approached to have their students participate in this study. Of these schools, ten agreed to participate. In the Netherlands, enrollment in special secondary education tailored for those with communication problems is regulated. Students are eligible only if a clinician or speech-language therapist indicates they have a (developmental) speech/language disorder, or if they have a formal diagnosis of hearing impairment from a certified audiologist. Additionally, seven mainstream secondary schools participated in this study. Within the students’ classrooms, participant recruitment took place through online video meetings or in-person meetings, during which the first author explained the research project to the participants. The class mentors of the students informed all parents via email or a paper letter that included a link to an infographic and a video explaining the research project supported by Sign Supported Dutch and Sign Language of the Netherlands. If students wanted to participate, they provided written, active, and informed consent. Since the age of consent in the Netherlands is 16, for students under 16 years old, additional written, active, and informed consent from their parents or guardians was required. The consent form was sign supported to DHH students who received education supported with Sign Language of the Netherlands or Sign Supported Dutch. To ensure consistency between interpreters, the following precautions were taken. A week before the test administration, the interpreters received the instrument questionnaires for preparation. They were scheduled for a half-hour meeting with the test administrator prior to the test to discuss any questions. The interpreters were instructed to adhere closely to the original questions without providing additional explanations. A simplified version of each question was prepared for the schemas and resilience questionnaires and was used when the adolescents needed further clarification. The only exclusion criterion was a nonverbal IQ score below 80; IQ scores were obtained from inspecting school records. Data collection consisted of four student questionnaires and took place between 2021 and 2023. The assessment duration was informed by consultations with special secondary schools, which recommended 60–120 min as a guideline for examinations. We ensured our testing battery adhered to these standards to avoid exceeding students’ cognitive load. The first author, a licensed and registered behavioral specialist, administered the data collection. All questionnaires were administered individually, with the questionnaires read aloud in spoken Dutch in a private room at the students’ schools. The entire test administration was supported by a hired, qualified, independent sign language interpreter if DHH students received education supported by Sign Language of the Netherlands or Sign Supported Dutch, or when parents or students preferred interpreting. Adolescents were offered a 10-euro reward for their participation. Before administering the questionnaires, the first author explained that they needed to follow the duty of professional secrecy. The participating students were informed that these confidentiality rules meant the information they provided would be kept confidential, except in cases involving signs of physical, emotional, or mental harm or distress. In these cases, professional standards required the first author to transfer responsibility for the student by subsequently involving the school counselor, requesting them to follow the protocol for domestic violence or child abuse. Students could opt out at any point during the test administration and still receive ten euros. Every student and the parents or guardians of students under 16 years old received a report containing the results of the participant’s questionnaires within a month of participation. This report also contained advice based on those results, contact information for professional healthcare services, and the contact information of the first author for any questions.

This study received approval from the Ethics Committee of the Faculty of Social Sciences of Radboud University.

### 2.4 Instruments

#### 2.4.1 General demographics

Participants provided general demographic information, including type of communication problems, gender, age, and educational background, via a written questionnaire.

#### 2.4.2 Resilience

Resilience was assessed using the Dutch version of the Brief Resilience Scale (BRS-NL) (Smith et al., 2008; Soer et al., 2019), which measures an individual’s perceived ability to bounce back or recover from stress. The BRS-NL has six items, with half being positively worded (e.g., “I tend to bounce back quickly after hard times”) and the other half negatively worded (e.g., “I have a hard time making it through stressful events”). Respondents rate each item on a 5-point Likert scale from 1 (completely disagree) to 5 (completely agree), with reverse scoring for negatively worded items. The total score is calculated by averaging the six items, resulting in a continuous score ranging from one to five. A higher score indicates greater perceived resilience. Additionally, a categorical score can be used; scores below 3.00 are considered “low,” scores above 4.30 are considered “high” (Smith et al., 2012). Previous studies have shown that the BRS provides valid and reliable scores among student samples (Smith et al., 2008). For the Dutch version’s internal consistency, a sample of residents from a rehabilitation unit in a nursing home was tested, yielding a Cronbach’s alpha of 0.83 (Leontjevas et al., 2014).

#### 2.4.3 Schemas

The adolescents’ schemas were assessed using the Young Schema Questionnaire Short Version (YSQ-SF) (van Vlierberghe et al., 2004; Young, 1998). The YSQ-SF is a 75-item self-report measure consisting of 15 scales that evaluate dysfunctional schemas, each represented by five items, such as “I don’t fit in” or “I feel that I’m not lovable.” The dysfunctional schemas include emotional deprivation, abandonment, mistrust/abuse, social isolation, defectiveness/shame, failure to achieve, functional dependence, vulnerability to harm, enmeshment, subjugation, self-sacrifice, emotional inhibition, unrelenting standards, entitlement, and insufficient self-control. Items are rated on a 6-point Likert scale ranging from 1 (completely untrue) to 6 (completely true). A scale score is calculated by dividing the total scale score by five, the number of items per scale, resulting in a continuous schema scale score from one to six. A higher score indicates a more dysfunctional schema. The scale scores have no official interpretation. It is recommended to interpret the highest scale scores (five and six) as indicative of schema problems (Schema Therapy Institute, 2021; Rijkeboer, 2010), or to consider a mean scale score of two or higher per scale as suggestive of potential schema problems (Rijkeboer, 2010). Additionally, a strong correlation was observed between the schema questionnaire and a psychopathology screening instrument when using the total scales score as a continuous variable (Young et al., 2003). Due to the absence of guidelines for interpreting scores and the arbitrary nature of choosing cutoff scores, this study used continuous total scores, with scores ranging from 1 to 6, the average of all items, for analysis. The internal consistency of the Dutch version of the YSQ-SF15 was good, with Cronbach’s alpha ranging from 0.76 (unrelenting standards) to 0.92 (defectiveness/shame and failure to achieve) (Pauwels et al., 2018).

#### 2.4.4 Theory of mind

To assess advanced ToM, the ToMotion (Smit et al., 2022) was used. The ToMotion measures how well a person interprets, adapts to, and verbally responds to enacted social situations on video. The ToMotion begins with one practice item followed by 12 test items. Each item includes three questions: a descriptive question, an explanatory question, and a personalized question, “What did you just see happen?,” “Why did X (person no.1) say this?,” “What would you do if you were Y (person no. 2)?” For each question, scores of 0, 1, or 2 points are awarded based on the adequacy of the interpretation and response to the enacted social situations; the item score ranges from 0 to 6. The scoring format includes explanatory examples of the minimal response conditions, with zero indicating the lowest adequacy and two indicating the highest. With a score range of 0–72, a higher ToMotion score signifies a more adequate interpretation and adaptation to social situations. The internal consistency is good, with a Cronbach’s alpha of 0.76 (Smit et al., 2022). The instrument is available in spoken Dutch and in Sign Language of the Netherlands (SLN). During this study’s assessment period, it remained unclear whether the spoken Dutch and SLN versions measured the ToM construct to the same extent. To ensure a consistent approach, the spoken version was administered to all adolescents. For DHH adolescents who received sign-supported test administration, accessibility was maintained by using their preferred interpreter, either Sign Language of the Netherlands or Sign Supported Dutch. Because dividing attention between the video and the interpreter can lead to information loss, DHH adolescents viewed the 12 videos twice: an initial viewing followed by a second viewing paired with their preferred sign support.

#### 2.4.5 Executive functions

The adolescents’ EFs were assessed using the Executive Functions Questionnaire (EFQ) for teachers (Scholte and van der Ploeg, 2019), completed by the adolescents’ class mentors. A single overall EFs scale provides the total EFs score, ranging from zero to 96. A higher score indicates more problems in the EFs. This scale is divided into two base scales, each with 12 items: Executive Learning Functions and Executive Behavior Functions. Both base scales are divided into two subscales of six items. The base scale, Executive Learning Functions, contains “working memory” and “mental flexibility.” The base scale, Executive Behavior Functions, contains “impulse control” and “emotion regulation.” In this study, the overall EF scales score was used for analysis. The EFQ has 24 items (e.g., “Is forgetful,” “Is easily distracted”) to be rated on a 5-point Likert scale from 0 to 4 (not or scarcely, occasionally, regularly, often, very often). The raw scores are compared to a norm sample to derive decile scores and categorical labels: normal, normal-high, subclinical, clinical, and clinical-high. The internal consistency is strong, with all Cronbach’s alphas exceeding 0.80. The test-retest reliability for the main and basic scales is approximately 0.90, and for the specific scales, it exceeds 0.80 (Scholte and van der Ploeg, 2019).

#### 2.4.6 Social support

The perceived social support of the adolescents was operationalized by mapping their social network through a semi-structured interview (GGZ Ecademy, 2017). Adolescents listed their prominent (nuclear) family members, best friends, and significant others (such as neighbors and teachers). For each listed person, the adolescents indicated whether they experienced much, normal, little, or no support. No measurement properties were available for this instrument. While the instrument captures various dimensions of social support, the final analysis was limited to the dimension corresponding to the instrument’s highest level of social support. This study used the raw scores (continuous) of the participants’ total number of family members, friends, and significant others they perceived as providing “much support,” resulting in a single variable called “social support.”

### 2.5 Statistical analysis

Statistical analyses were performed using SPSS versions 29 and 30 (IBM Corp, 2023). *Z*-tests were employed to examine demographic differences among participants. The primary study outcome for the first research question focused on resilience in adolescents with CP. Before analyzing this question, the reliability of the resilience instrument (BRS) was assessed with Cronbach’s alpha. Two-sided *t*-tests were used due to the exploratory approach taken, comparing the reference group to adolescents with CP and comparing DHH adolescents with adolescents with DLD on resilience. A two-sided *z*-test was used to compare the proportions of adolescents reporting low resilience scores.

Regarding the second and third research questions, the descriptive statistics included percentages, means, and standard deviations of continuous variables: dysfunctional schemas, ToM, EFs, and social support. Two-sided *t*-tests were performed to compare DHH adolescents with adolescents with DLD on dysfunctional schemas, ToM, EFs, and social support. A two-sided *z*-test for proportions was used to compare groups on the proportion of clinical EFs scores. Before performing a multiple linear regression, the relationship between the four factors (dysfunctional schemas, ToM, EFs, social support) was tested using a bivariate correlation, and the test assumptions for performing a linear regression were tested. Two multiple linear regression analyses, one with and one without controlling for adolescents’ type of communication problems (DHH vs. DLD), were conducted to examine the contributions of dysfunctional schemas, ToM, EFs, and social support to resilience, using continuous scores.

## 3 Results

### 3.1 Participant groups

Table 1 shows the gender distribution and educational attainment for the groups: adolescents with CP compared to the reference group, and DHH adolescents compared to adolescents with DLD. These groups were comparable in terms of gender distribution, but the educational attainment showed a few significant differences. A larger proportion of the reference group participated in a more theoretical educational program than adolescents with CP, while a larger proportion of adolescents with CP engaged in a more practical educational program than the reference group. Within the group of adolescents with CP, analysis showed that a significantly larger proportion of DHH adolescents participated in a more theoretical educational program compared to adolescents with DLD. Although these differences were observed, covariates were not statistically controlled for, as doing so would violate the assumption of independence between the covariate and the grouping variable (Field, 2024).

### 3.2 Resilience

The reliability of the resilience instrument (BRS) was considered good, with a Cronbach’s alpha of 0.84 (see Supplementary Table 3). An independent-samples *t*-test was conducted to compare resilience between groups (see Supplementary Table 4). The results reveal that adolescents with CP (*M* = 2.85, *SD* = 0.74) reported significantly lower resilience than the reference group (*M* = 3.58, *SD* = 0.86) [*t*_(211)_ = 6.57, two-sided *p* < 0.001]. When comparing adolescents in reporting low resilience scores, the proportion of adolescents with CP was significantly higher than that of the reference group (two-sided *p* < 0.001). Within the group of adolescents with CP, DHH adolescents (*M* = 2.98, *SD* = 0.71) and adolescents with DLD (*M* = 2.80, *SD* = 0.75) were compared. No significant differences were found in their reported resilience [*t*_(125)_ = −1.23, two-sided *p* > 0.1). Similarly, among adolescents with CP, no proportion differences were found between DHH adolescents and those with DLD in reporting low resilience scores (*p* > 0.05) (see Supplementary Table 6). The proportion of adolescents with CP reporting low resilience scores compared to their peers in mainstream education seems noteworthy, with over 50% reporting low resilience: RG 20.9%, DHH 53.1%, DLD 57.9%.

### 3.3 Factors predicting resilience

Before testing the predictive values of dysfunctional schemas, ToM, EFs, and social support to resilience, descriptive statistics and group comparisons for these factors were examined (see Supplementary Tables 7–16).

The comparison of dysfunctional schemas within the group of adolescents with CP indicated no differences between groups [*t*_(125)_ = 0.94, two-sided *p* > 0.1]. Similarly, when comparing ToM within the group of adolescents with CP no differences were found [*t*_(125)_ = 0.33, two-sided *p* > 0.1]. Furthermore, the comparison of EFs within the group of adolescents with CP, indicated no differences [*t*_(125)_ = 0.62, two-sided *p* > 0.1], also no differences were found when comparing the proportion of clinical scores on EFs between DHH adolescents and those with DLD (*z* = 0.140, two-sided *p* > 0.1). Among the adolescents with CP, 88% reported non-clinical EFs scores. Lastly, the comparison of social support within the group of adolescents with CP revealed no differences [*t*_(125)_ = 0.58, two-sided *p* > 0.1].

Before testing the primary outcomes of the second and third research questions, the correlations among dysfunctional schemas, ToM, EFs, and social support were examined and found to be weak and nonsignificant (all *r*’s < 0.15, *p*’s > 0.1) (see Supplementary Table 17). Additionally, the test assumptions for performing a multiple linear regression were met (see Supplementary Charts 1, 2 and Supplementary Tables 18–20). A multiple linear regression analysis was performed with these four factors entered simultaneously to predict resilience (see Supplementary Table 21). The four factors, dysfunctional schemas, ToM, EFs, and social support, explained a substantial proportion (33.6%) of the variance in resilience. The ANOVA indicated that the overall regression model was statistically significant (*F* = 15.45, *p* < 0.001). In response to the research question about the extent to which the four factors contribute to resilience, ToM, EFs, and social support did not appear to contribute significantly, with *p*-values of 0.322, 0.064, and 0.407, respectively. Dysfunctional schemas were the only significant predictor of resilience (*t* = −7.62, *p* < 0.001). The standardized beta coefficient of −0.57 indicates a strong negative relationship, meaning greater schema problems are associated with lower resilience.

### 3.4 The moderating role of type of communication problems in predicting resilience

To determine whether having hearing loss or having DLD influences the contribution of dysfunctional schemas, ToM, EFs, and social support to resilience, it was examined whether adolescents’ type of communication problems moderates these contributions (see Supplementary Table 22). Regression analyses with nine variables (type of communication problems, dysfunctional schemas, ToM, EFs, social support, and the interaction effects of type of communication problems with dysfunctional schemas, ToM, EFs, and social support) revealed that these variables accounted for approximately 38.8% of the variance in resilience. The ANOVA indicated that, again, the overall regression model was statistically significant (*F* = 8.25, *p* < 0.001). The main effects of dysfunctional schemas again reached significance (*t* = −5.32, *p* < 0.001). Due to the addition of variables in this regression analysis the ToM main effect reached significance (*t* = −2.39, *p* < 0.05). Importantly, the relationship between ToM and resilience was moderated by type of communication problems (*t* = 2.73, *p* < 0.05). The plot showed no significant relationship between adolescents with DLD and ToM, but a negative relationship indicating that as DHH adolescents’ ToM abilities increased, their resilience decreased (see Supplementary Chart 3).

## 4 Discussion

The results of this study indicate that adolescents with CP in special secondary education show significantly less resilience than adolescents without CP in mainstream education. Half of the adolescents with CP reported low resilience scores, while approximately one-fifth of the mainstream controls did. The nature of their communication problems, whether having hearing loss or having DLD, did not significantly affect their resilience. To start understanding resilience predictors, four factors that contribute to resilience were examined for their impact: dysfunctional schemas, ToM, EFs, and social support. No significant differences were observed between DHH adolescents and those with DLD regarding these factors. Contrary to this study’s expectations, the multiple linear regression analysis showed no significant relationships between these factors and resilience, except for dysfunctional schemas. This suggests that dysfunctional schemas of adolescents with CP are the sole significant predictor of their resilience. An unexpected finding emerged when evaluating the moderating effect of the type of communication problems on the relationship between the four factors and resilience. A negative relationship was found in DHH adolescents, as their ToM abilities increased, their resilience decreased. In contrast, no relationship was found for adolescents with DLD.

The hypothesis that the resilience of adolescents with CP would be compromised was confirmed. Differences in resilience between typically developing adolescents and those with CP were significant. Not only did adolescents with CP report substantially lower resilience scores, but the proportion of adolescents with CP reporting low resilience was nearly three times (2.7) higher than that of their typically developing peers. This reflects a notable and concerning disparity, indicating that this group of youth is particularly vulnerable and may remain so, with less resilience; many of these adolescents appear insufficiently equipped to handle future stressors. The educational setting may play a role. Students in specialized settings might represent a more vulnerable subgroup; therefore, these findings cannot be generalized to all youth with CP. Among the four factors examined, an exploratory approach was taken toward social support, while dysfunctional schemas, ToM, and EFs were hypothesized to predict resilience. Dysfunctional schemas emerged as the only predictor of resilience in this group of adolescents with CP.

The hypothesis that ToM predicts resilience was not confirmed in this study. A possible explanation may lie in the complexity of assessing ToM as a construct, as crucial aspects of its development and underlying mechanisms are still poorly understood (Rakoczy, 2022). The ToM instrument used in this study is an elaborate tool assessing ToM functions appropriate for adolescents. Even so, the functions evaluated are a simplification of reality; the videos feature the same two people encountering one dilemma per video, and participants do not have a time limit to respond. In real life, navigating adolescence involves evolving social and emotional interactions, including increased parental expectations for independence and more complex social demands as peer interactions become more intricate (Crone et al., 2022; Sisk and Gee, 2022). In these multifaceted situations, interactions are likely to require advanced ToM skills, including the ability to consider multiple interests simultaneously within a short timeframe, which current ToM assessments cannot measure. Thus, this could serve as an important focal point for future research.

However, a moderating effect of the type of communication problems on the relationship between ToM and resilience was observed, which indicated a negative relationship between ToM and resilience only in DHH adolescents. This observation appears to be incidental. To assess the integrity of the data with respect to this finding, two statistical control analyses were performed. Cook’s distance analyses identified only four deviating cases. Careful inspection of these cases did not justify their exclusion from the data file. Subsequent percentile bootstrapping confirmed that the results remain robust and consistent and are not driven by outliers (see Supplementary Table 23). Although we cannot provide a definitive explanation, we have no evidence to suggest that the finding is a measurement artifact. Consequently, further research involving a larger group of DHH participants is warranted to validate this finding.

Contrary to the findings of this study, a predictive relationship between EFs and resilience was expected based on prior studies by Mecha et al. (2024), Morgan and Dye (2020), Niu et al. (2024), and Yule et al. (2019). However, a closer look at the referenced studies revealed differences in the instruments and informants used to assess EFs across those studies and the present study. In the present study, a deliberate decision was made to have the EFs assessment completed by participants’ class mentors rather than their parents or guardians to reduce bias and potential language barriers. As a consequence, important information about EFs might be missing for two reasons. First, class mentors in secondary education only teach their mentor class for a limited number of hours each week. Second, their knowledge of EFs is confined to the school setting, and they may not have a complete picture of the student’s overall executive functioning, especially in more stressful contexts like doing homework, playing games, or going on trips. Also, students may demonstrate better EFs in a structured school environment. Thus, our choice of class mentors as informants of adolescents’ EFs may have affected our results. Although no significant relationship was found between EFs and resilience in this study, this does not imply that EFs do not contribute to adaptive outcomes. The multifaceted nature of both constructs may require alternative measurement approaches to fully capture their interaction. Future studies should therefore include multiple perspectives and a more complete range of EFs measures.

This study also found no relationship between resilience and social support, as expected based on decades of research (Cohen and Wills, 1985; Masten, 2018; Vila, 2021; Yule et al., 2019; Zalta et al., 2021). Here, a possible explanation may lie in how this relationship was assessed in these studies. The construct of resilience was often not measured directly; instead resilience was presented as a framework but not empirically tested (Masten, 2016, 2018; Masten et al., 2021) or assessments made under the umbrella of “resilience” frequently captured related factors like the impact of stress or specific stress symptoms such as posttraumatic stress disorder (Cohen and Wills, 1985; Vila, 2021; Yule et al., 2019; Zalta et al., 2021). These studies may not have truly measured “resilience” but rather a proxy or related outcome, thereby failing to capture the true influence of social support on resilience itself, which implies that the indicated long-standing relationship may be less valid.

The results of this study suggest that adolescents with CP in special secondary education are a vulnerable group of youth. They show significantly lower resilience compared to adolescents without CP in mainstream education. The complexity of disentangling factors that contribute to the dynamic construct of resilience appears considerable. When examining the predictive value of four factors, schemas, ToM, EFs, and social support, only schemas emerged as a significant predictor of resilience, indicating that more dysfunctional schemas are associated with less resilience.

## 5 Limitations and recommendations

Because this study focused on adolescents enrolled in special secondary education (in the Netherlands 50% of all DHH students), the extent to which the results apply to adolescents with CP in mainstream education is unknown. More research is needed to determine whether similar problems in developing resilience are present or even more pronounced among students with CP in mainstream education, or whether this subgroup is an exception, given the severity and implications of the observed outcomes.

In this study, we decided not to exclude participants with comorbid conditions, such as ASD and ADHD. This may have had some impact on results across EFs and ToM measures. Further research is needed to determine whether resilience and potentially contributing components differ in these subgroups compared to adolescents with CP without comorbid conditions.

The unbalanced distribution of group sample sizes (DHH adolescents *n* = 32 and adolescents with DLD *n* = 95), particularly in the regression outcomes, may primarily reflect the profile of adolescents with DLD and may mask effects specific to DHH adolescents, thereby limiting the ability to draw definitive conclusions about them.

Another potential limitation of the study is the risk of common-method bias. While EFs and ToM were evaluated using objective measures, schemas, resilience, and social networks were assessed via self-report questionnaires. Therefore, the observed relationships should be interpreted with caution.

Resilience appears to be influenced by many factors. This study contributes to research on the resilience of adolescents with CP. The findings of the current study can serve as a foundation for future research into communication-related factors, such as communication access, classroom participation, and adverse communication experiences.

Analysis of the resilience data reveals a substantial difference in resilience between adolescents with CP in special education and adolescents without CP in mainstream education. This difference has important implications. Since resilience involves maintaining mental stability during or after exposure to (cumulative) life stressors and effectively adapting to life’s challenges (Center of the Developing Child, 2025; Masten, 2016, 2018; Shafi et al., 2020), the low resilience scores among adolescents with CP in special education suggest an ongoing disadvantage in coping with new adverse situations. As resilience is considered a dynamic ability, having limited resilience to cope with current adversities may reinforce a less effective stress response, making these adolescents even more vulnerable to future stressors (Boyce et al., 2021). Since this study identified a predictive relationship between adolescents’ schemas and their resilience, it is recommended to support them by strengthening healthy perceptions of themselves, others, situations, and the world. This could include efforts to improve the parent-child relationship, given that dysfunctional schemas often stem from unmet emotional needs during childhood, with the earliest schemas being the strongest and typically originating within the nuclear family (Young et al., 2003). Throughout development, attention can be placed on fostering a positive self-image, while schools can promote inclusion and acceptance, for example, through anti-bullying programs.

## Data Availability

The datasets presented in this study can be found in online repositories. The names of the repository/repositories and accession number(s) can be found here, https://doi.org/10.34973/wkab-ja28 and in the article/Supplementary material.
