# Supplementary material for: Predictors of resilience in deaf and hard of hearing adolescents and adolescents with developmental language disorders
Source: Front Psychol. 2026 Jun 26;17:1773481. doi: 10.3389/fpsyg.2026.1773481 (PMC13349397; doi:10.3389/fpsyg.2026.1773481)
Supplement: Supplementary file 1 [file Supplementary_file_1.docx]

***Supplementary material***

**Table 1**

*Characteristics of DHH Participants*

|  | ***n* DHH Participants** |
| --- | --- |
| **Hearing loss** |  |
| Mild 26-40 dB (HL) | 4 |
| Moderate 41-70 dB (HL) | 13 |
| Severe 71-90 dB (HL) | 6 |
| Profound >91 dB (HL) | 9 |
| **Technological support** |  |
| One cochlear implant | 2 |
| One hearing aid | 4 |
| Two cochlear implants | 3 |
| Two hearing aids | 17 |
| Mixed cochlear implant & hearing aid | 6 |

Note: *N* = 32

**Table 2**

*Robust Tests of Equality of Means DHH Adolescents versus Adolescents with DLD*

|  |  | Statistic^a^ | df1 | df2 | Sig. |
| --- | --- | --- | --- | --- | --- |
| Resilience total | Welch | 1.504 | 1 | 55.729 | .225 |
| Schema total | Welch | .883 | 1 | 62.389 | .351 |
| ToM score | Welch | .110 | 1 | 56.202 | .741 |
| EFs | Welch | .382 | 1 | 43.952 | .540 |
| EFs learning functions | Welch | .002 | 1 | 45.477 | .965 |
| EFs behavior functions | Welch | 1.507 | 1 | 45.259 | .226 |
| Much support total | Welch | .335 | 1 | 49.677 | .565 |

Note: DHH *n* = 32, DLD *n* = 95. a. Asymptotically *F* distributed

**Table 3**

*Reliability Resilience Instrument - BRS*

| Reliability  BRS | Cronbach's alpha | Cronbach's alpha standardized items | *N* of items |
| --- | --- | --- | --- |
|  | .840 | .841 | 6 |

Note: *N* = 213. Reference group *n* = 86. Adolescents with CP *n* = 127.

**Table 4**

*Comparing Resilience Scores*

| Participants | RG | | CP | | Two-sided *p* | *t* | 95% *CI* |
| --- | --- | --- | --- | --- | --- | --- | --- |
|  | *M* | *SD* | *M* | *SD* |  |  |  |
| Resilience | 3.58 | .86 | 2.85 | .74 | <.001** | 6.569 | [.5, 1.0] |
| Participants | DHH | | DLD | | Two-sided *p* | *t* | 95% *CI* |
|  | *M* | *SD* | *M* | *SD* |  |  |  |
| Resilience | 2.98 | .71 | 2.80 | .75 | .225 | -1.226 | [-.1, .5] |

Note: Equal variances not assumed. *N* = 213. Reference group, RG *n* = 86. Adolescents with CP, CP *n* = 127. DHH adolescents *n* = 32, adolescents with DLD *n* = 95. ***p* < .001.

**Table 5**

*Resilience Group Percentages*

|  | Resilience | Low | Normal | High | Total |
| --- | --- | --- | --- | --- | --- |
| Groups |  |  |  |  |  |
| RG | Count | 18 | 51 | 17 | 106 |
|  | % within group | 20.93 | 59.30 | 19.77 | 100.00 |
| CP | Count | 72 | 51 | 4 | 127 |
|  | % within group | 56.69 | 40.16 | 3.15 | 100.00 |
| DHH | Count | 17 | 14 | 1 | 32 |
|  | % within group | 53.13 | 43.75 | 3.13 | 100.00 |
| DLD | Count | 55 | 37 | 3 | 95 |
|  | % within group | 57.89 | 38.95 | 3.16 | 100.00 |

Note: *N* = 213. Reference group, RG *n* = 86. Adolescents with CP, CP *n* = 127.

DHH *n* = 32, DLD *n* = 95.

**Table 6**

*Independent Samples Proportion Test Low Resilience Scores*

| Groups | Proportion | Proportion Difference | *z* | Two-sided *p* | 95% *CI* |
| --- | --- | --- | --- | --- | --- |
| RG | .209 |  |  |  |  |
| CP | .567 | -.358 | -5.184 | <.001** | [-.5, -.2] |
| DHH | .531 |  |  |  |  |
| DLD | .579 | -.048 | -.471 | .638 | [-.2, .2] |

Note: *N* = 213. Reference group, RG *n* = 86. Adolescents with CP, CP *n* = 127.

DHH *n* = 32, DLD *n* = 95. ***p* < .001.

**Table 7**

*Reliability Schema Instrument - YSQ-SF*

| Reliability  YSQ-SF | Cronbach's alpha | Cronbach's alpha standardized items | *N* of scales |
| --- | --- | --- | --- |
|  | .920 | .922 | 15 |

Note: *N* = 127 (DHH *n* = 32, DLD *n* = 95).

**Table 8**

*Descriptives Schema Score - Adolescents with CP*

| Participants | CP | | Minimum | Maximum | Variance |
| --- | --- | --- | --- | --- | --- |
|  | *M* | *SD* |  |  |  |
| Total schema score | 2.48 | .65 | 1.13 | 4.19 | .427 |

Note: Adolescents with CP, CP *N* = 127 (DHH *n* = 32, DLD *n* = 95).

**Table 9**

*Comparing Schemas - DHH Adolescents and Adolescents with DLD*

| Participants | DHH | | DLD | | *t* | Two-sided *p* | 95% *CI* |
| --- | --- | --- | --- | --- | --- | --- | --- |
| Schemas | *M* | *SD* | *M* | *SD* |  |  |  |
| Total schemas | 2.39 | .58 | 2.51 | .68 | -.940 | .351 | [-.4, .1] |

Note: Equal variances not assumed. *N* = 127. DHH *n* = 32, DLD *n* = 95.

**Table 10**

*Descriptives ToM - Adolescents with CP*

| Participants | CP | | Minimum | Maximum | Variance |
| --- | --- | --- | --- | --- | --- |
|  | *M* | *SD* |  |  |  |
| ToM score | 47.37 | 7.04 | 24 | 61 | 49.584 |

Note: Adolescents with CP, CP *N* = 127 (DHH *n* = 32, DLD *n* = 95).

**Table 11**

*Comparing ToM - DHH Adolescents and Adolescents with DLD*

| Participants | DHH | | DLD | | *t* | Two-sided *p* | 95% *CI* |
| --- | --- | --- | --- | --- | --- | --- | --- |
| ToM | *M* | *SD* | *M* | *SD* |  |  |  |
|  | 47.72 | 6.76 | 47.25 | 7.16 | .332 | .741 | [-2.4, 3.3] |

Note: Equal variances not assumed. *N* = 127. DHH *n* = 32, DLD *n* = 95.

**Table 12**

*Descriptives Executive Functions - Adolescents with CP*

|  | | CP | | Minimum | Maximum | Variance |
| --- | --- | --- | --- | --- | --- | --- |
|  | | *M* | *SD* |  |  |  |
| **Total Executive Functions** | | 23.55 | 18.65 | 0 | 84 | 347.789 |
| Learning functions | | 13.76 | 10.01 | 0 | 43 | 100.166 |
|  | Working memory | 7.80 | 5.88 | 0 | 22 | 34.572 |
|  | Mental Flexibility | 5.96 | 4.68 | 0 | 23 | 21.864 |
| Behavior functions | | 9.79 | 10.02 | 0 | 43 | 100.359 |
|  | Impulse inhibition | 5.74 | 6.01 | 0 | 23 | 36.067 |
|  | Emotion regulation | 4.05 | 4.84 | 0 | 23 | 23.410 |

Note: Adolescents with CP, CP *N* = 127 (DHH *n* = 28, DLD *n* = 86).

**Table 13**

*Comparing Executive Functions - DHH Adolescents and Adolescents with DLD*

| Participants | DHH | | DLD | | *t* | Two-sided *p* | 95% *CI* |
| --- | --- | --- | --- | --- | --- | --- | --- |
|  | *M* | *SD* | *M* | *SD* |  |  |  |
| EFs | 25.56 | 22.48 | 22.87 | 17.25 | .618 | .540 | [-6.1, 11.5] |
| Learning functions | 13.69 | 11.67 | 13.79 | 9.45 | -.045 | .965 | [-4.7, 4.5] |
| Behavior functions | 11.88 | 11.65 | 9.08 | 9.37 | 1.228 | .226 | [-1.8, 7.4] |

Note: Equal variances not assumed. *N* = 127. DHH *n* = 32. DLD *n* = 95.

**Table 14**

*Proportions Clinical EFs Scores*

| Groups | Proportion |  |  |  |  |
| --- | --- | --- | --- | --- | --- |
| CP | .118 |  |  |  |  |
|  |  | Proportion Difference | *z* | Two-sided *p* | 95% *CI* |
| DHH | .125 |  |  |  |  |
| DLD | .116 | .009 | .140 | .889 | [-.1, .2] |

Note: Adolescents with CP, CP *N* = 127. DHH *n* = 28, DLD *n* = 86.

**Table 15**

*Descriptives Number of People Providing Much Support - Adolescents with CP*

| Participants | CP | | Minimum | Maximum | Variance |
| --- | --- | --- | --- | --- | --- |
|  | *M* | *SD* |  |  |  |
| People providing  much support | 4.85 | 3.30 | 0 | 14 | 10.890 |

Note: Adolescents with CP, CP *N* = 127 (DHH *n* = 32, DLD *n* = 95).

**Table 16**

*Comparing Number of People Providing Much Support - DHH Adolescents and*

*Adolescents with DLD*

| Participants | DHH | | DLD | | *t* | Two-sided *p* | 95% *CI* |
| --- | --- | --- | --- | --- | --- | --- | --- |
|  | *M* | *SD* | *M* | *SD* |  |  |  |
| People providing  much support | 5.16 | 3.53 | 4.75 | 3.23 | .579 | .565 | [-1.0, 1.8] |

Note: Equal variances not assumed*. N* = 127. DHH *n* = 32, DLD *n* = 95.

**Table 17**

*Correlations Social Support, Schemas, ToM, EFs - DHH Adolescents and Adolescents*

*with DLD*

| Correlations | | Support | Schemas | ToM | EFs |
| --- | --- | --- | --- | --- | --- |
| Social support | Pearson Correlation | 1 | -.065 | .122 | -.003 |
|  | Sig. (2-tailed) |  | .468 | .173 | .975 |
|  | *N* | 127 | 127 | 127 | 127 |
| Schemas | Pearson Correlation | -.065 | 1 | -.081 | .076 |
|  | Sig. (2-tailed) | .468 |  | .368 | .396 |
|  | *N* | 127 | 127 | 127 | 127 |
| ToM | Pearson Correlation | .122 | -.081 | 1 | -.120 |
|  | Sig. (2-tailed) | .173 | .368 |  | .178 |
|  | *N* | 127 | 127 | 127 | 127 |
| EFs | Pearson Correlation | -.003 | .076 | -.120 | 1 |
|  | Sig. (2-tailed) | .975 | .396 | .178 |  |
|  | *N* | 127 | 127 | 127 | 127 |

Note: *N* = 127 (DHH *n* = 32, DLD *n* = 95).

**Chart 1**

*Normal P-P Plot of Regression Standardized Residual - Dependent Variable Resilience Total - DHH Adolescents and Adolescents with DLD*


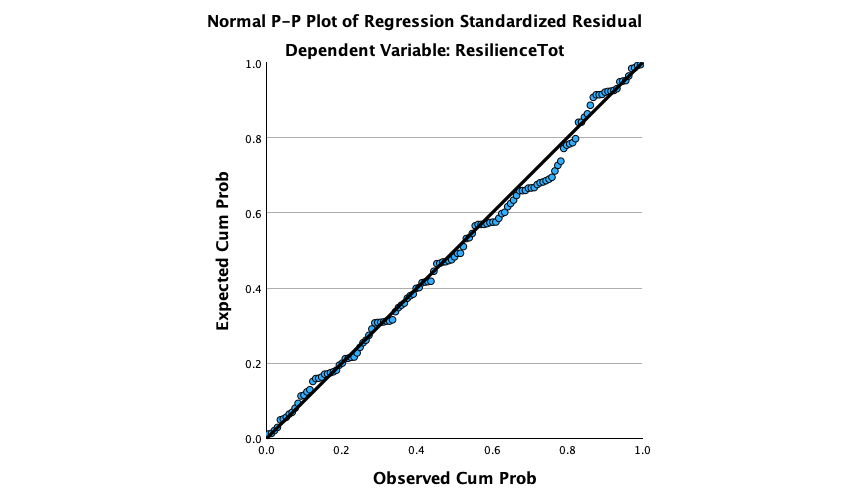


Note: *N* = 127 (DHH *n* = 32, DLD *n* = 95).

**Chart 2**

*Scatterplot - Dependent Variable Resilience Total - DHH Adolescents and Adolescents with DLD*


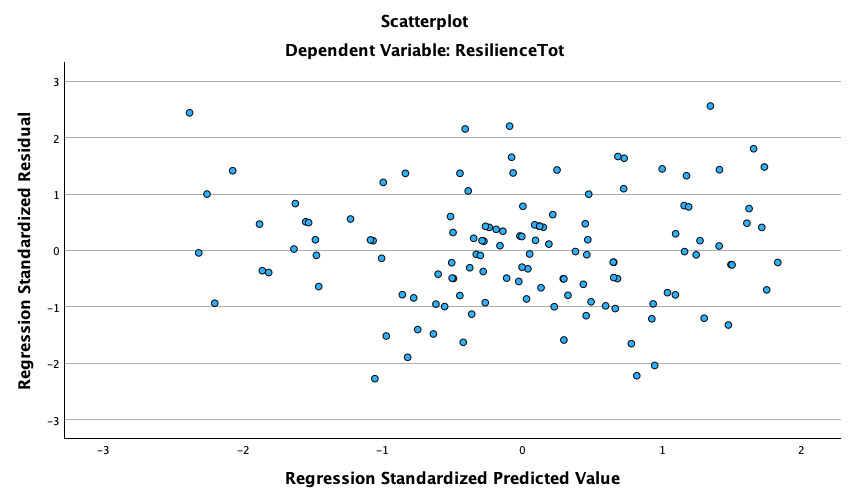


Note: *N* = 127 (DHH *n* = 32, DLD *n* = 95).

**Table 18**

*Normality Descriptives - DHH Adolescents and Adolescents with DLD*

|  |  |  | Statistic | Std.error |
| --- | --- | --- | --- | --- |
| Unstandardized residual | Mean |  | .0000000 | .05353032 |
|  | 95% Confidence interval for mean | Lower bound | -.1059349 |  |
|  |  | Upper bound | .1059349 |  |
|  | 5% trimmed mean |  | -.0057472 |  |
|  | Median |  | -.0118407 |  |
|  | Variance |  | .364 |  |
|  | Std. deviation |  | .60325610 |  |
|  | Minimum |  | -1.35624 |  |
|  | Maximum |  | 1.55143 |  |
|  | Range |  | 2.90767 |  |
|  | Interquartile range |  | .78427 |  |
|  | Skewness |  | .197 | .215 |
|  | Kurtosis |  | -.107 | .427 |
| Standardized residual | Mean |  | .0000000 | .08731579 |
|  | 95% Confidence interval for mean | Lower bound | -.1727954 |  |
|  |  | Upper bound | .1727954 |  |
|  | 5% trimmed mean |  | -.0093745 |  |
|  | Median |  | -.0193140 |  |
|  | Variance |  | .968 |  |
|  | Std. deviation |  | .98399897 |  |
|  | Minimum |  | -2.21223 |  |
|  | Maximum |  | 2.53060 |  |
|  | Range |  | 4.74283 |  |
|  | Interquartile range |  | 1.27926 |  |
|  | Skewness |  | .197 | .215 |
|  | Kurtosis |  | -.107 | .427 |

Note: *N* = 127 (DHH *n* = 32, DLD *n* = 95).

**Table 19**

*Explore Normality - Case Processing Summary - DHH Adolescents and Adolescents with DLD*

|  | Valid | | Missing | | Total | |
| --- | --- | --- | --- | --- | --- | --- |
|  | *N* | Percent | *N* | Percent | *N* | Percent |
| Unstandardized Residual | 127 | 100.0% | 0 | 0.0% | 127 | 100.0% |
| Standardized Residual | 127 | 100.0% | 0 | 0.0% | 127 | 100.0% |

Note: *N* = 127 (DHH *n* = 32, DLD *n* = 95).

**Table 20**

*Explore Normality - Tests of Normality - DHH Adolescents and Adolescents with DLD*

|  | Kolmogorov-Smirnov^a^ | | | Shapiro-Wilk | | |
| --- | --- | --- | --- | --- | --- | --- |
|  | Statistic | *df* | Sig. | Statistic | *df* | Sig. |
| Unstandardized Residual | .070 | 127 | .200^*^ | .990 | 127 | .503 |
| Standardized Residual | .070 | 127 | .200^*^ | .990 | 127 | .503 |

Note: *N* = 127 (DHH *n* = 32, DLD *n* = 95). *This is a lower bound of the true significance.

a. Lilliefors Significance Correction

**Table 21**

*Regression ToM, EFs, Schemas, and Social Support to Resilience - DHH Adolescents and Adolescents with DLD*

| **Model summary^b^** | *R* | *R* square | Adjusted *R* square | Std. error of the estimate |
| --- | --- | --- | --- | --- |
|  | .580^a^ | .336 | .314 | .61307 |

Note: *N* = 127 (DHH *n* = 32, DLD *n* = 95). a. Predictors: (constant), social support, schemas, EFs, ToM. b. Dependent: Total resilience score.

| **ANOVA^a^** | Sum of squares | *df* | Mean square | *F* | Sig. |
| --- | --- | --- | --- | --- | --- |
| Regression | 23.226 | 4 | 5.806 | 15.449 | <.001^b**^ |
| Residual | 45.854 | 122 | .376 |  |  |
| Total | 69.080 | 126 |  |  |  |

Note: *N* = 127 (DHH *n* = 32, DLD *n* = 95). a. Dependent variable: Total resilience score. b. Predictors: (constant), social support, schemas, EFs, ToM. ***p* < .001.

| **Coefficients^a^** | Unstandardized Coefficients | | Standardized Coefficients | *t* | Sig. |
| --- | --- | --- | --- | --- | --- |
|  | *B* | Std. Error | Beta |  |  |
| (Constant) | 4.611 | .454 |  | 10.153 | <.001 |
| Social support | .014 | .017 | .062 | .831 | .407 |
| Schemas | -.641 | .084 | -.566 | -7.619 | <.001** |
| ToM | -.008 | .008 | -.075 | -.994 | .322 |
| EFs | .006 | .003 | .139 | 1.869 | .064 |

Note: *N* = 127 (DHH *n* = 32, DLD *n* = 95). a. Dependent variable: Total resilience score. ***p* < .001.

| **Residuals statistics^a^** | Minimum | Maximum | Mean | Std. Deviation | *N* |
| --- | --- | --- | --- | --- | --- |
| Predicted Value | 1.8258 | 3.6090 | 2.8491 | .42934 | 127 |
| Residual | -1.35624 | 1.55143 | .00000 | .60326 | 127 |
| Std. Predicted Value | -2.383 | 1.770 | .000 | 1.000 | 127 |
| Std. Residual | -2.212 | 2.531 | .000 | .984 | 127 |

Note: *N* = 127 (DHH *n* = 32, DLD *n* = 95). a. Dependent variable: Total resilience score.

**Table 22**

*Interaction of Type of Communication Problems on Regression of ToM, EFs, Schemas, and Social Support to Resilience*

| **Variables Entered** | Variables Removed | Method |
| --- | --- | --- |
| TCP, ToM, EFs, Schemas, Social support  ToM x TCP  EFs x TCP  Schemas x TCP  Social support x TCP^b^ | . | Enter |

Note: *N* = 127 (DHH *n* = 32, DLD *n* = 95). TCP = Type of communication problems. a. Dependent variable: Total resilience. b. All requested variables entered.

| **Model summary^b^** | *R* | *R* square | Adjusted *R* square | Std. error of the estimate |
| --- | --- | --- | --- | --- |
|  | .623^a^ | .388 | .341 | .60104 |

Note: *N* = 127 (DHH *n* = 32, DLD *n* = 95. a. Predictors: (Constant), TCP, ToM x TCP, Social support, Schemas x TCP, EFs x TCP, EF, Social support x TCP, ToM, Schemas. b. Dependent variable: Total resilience.

| **ANOVA^a^** | Sum of squares | *df* | Mean square | *F* | Sig. |
| --- | --- | --- | --- | --- | --- |
| Regression | 26.813 | 9 | 2.979 | 8.247 | <.001^b**^ |
| Residual | 42.267 | 117 | .361 |  |  |
| Total | 69.080 | 126 |  |  |  |

Note: *N* = 127 (DHH *n* = 32, DLD *n* = 95. a. Dependent variable: Total resilience. b. Predictors: (Constant), TCP, ToM x TCP Social support, Schemas x TCP, EFs x TCP, EFs, Social support x TCP, ToM, Schems. ***p* < .001.

| **Coefficients^a^** | Unstandardized Coefficients | | Standardized Coefficients | *t* | Sig. |
| --- | --- | --- | --- | --- | --- |
|  | *B* | Std. Error | Beta |  |  |
| (Constant) | 2.891 | .063 |  | 46.035 | <.001** |
| SupportCentered | .007 | .018 | .033 | .399 | .691 |
| SchemasCentered | -.599 | .113 | -.528 | -5.319 | <.001** |
| ToMCentered | -.022 | .009 | -.211 | -2.393 | .018* |
| EFsCentered | .004 | .003 | .098 | 1.204 | .231 |
| Social support x TCP | .016 | .018 | .073 | .895 | .373 |
| Schemas x TCP | -.026 | .113 | -.023 | -.234 | .815 |
| ToM x TCP | .025 | .009 | .241 | 2.729 | .007* |
| EFs x TCP | .003 | .003 | .084 | 1.030 | .305 |
| TCP | -.062 | .063 | -.073 | -.984 | .327 |

Note: *N* = 127 (DHH *n* = 32, DLD *n* = 95). a. Dependent variable: Total resilience score. Predictors (constant), controlled for type of communication problems (TCP). ToM x TCP, EFs x TCP, Schemas x TCP, Social support x TCP. **p* < .05. ***p* < .001.

| **Residual Statistics^a^** |  | |  |  |  |
| --- | --- | --- | --- | --- | --- |
|  | Minimum | Maximum | Mean | *SD* | *N* |
| Predicted Value | 1.7945 | 4.1860 | 2.8491 | .46130 | 127 |
| Std. Predicted Value | -2.286 | 2.898 | .000 | 1.000 | 127 |
| Standard Error of Predicted Value | .078 | .341 | .157 | .061 | 127 |
| Adjusted Predicted Value | 1.6596 | 4.3119 | 2.8438 | .46849 | 127 |
| Residual | -1.15290 | 1.55092 | .00000 | .57918 | 127 |
| Std. Residual | -1.918 | 2.580 | .000 | .964 | 127 |
| Stud. Residual | -1.987 | 2.681 | .004 | 1.010 | 127 |
| Deleted Residual | -1.31880 | 1.88142 | .00527 | .63868 | 127 |
| Stud. Deleted Residual | -2.013 | 2.756 | .005 | 1.018 | 127 |
| Mahal. Distance | 1.103 | 39.568 | 8.929 | 8.342 | 127 |
| Cook's Distance | .000 | .267 | .011 | .032 | 127 |
| Centered Leverage Value | .009 | .314 | .071 | .066 | 127 |

Note: *N* = 127 (DHH *n* = 32, DLD *n* = 95. a. Dependent variable: Total resilience score

**Chart 3**

*Simple Scatter Relationship ToM - Resilience - DHH Adolescents and Adolescents with DLD*


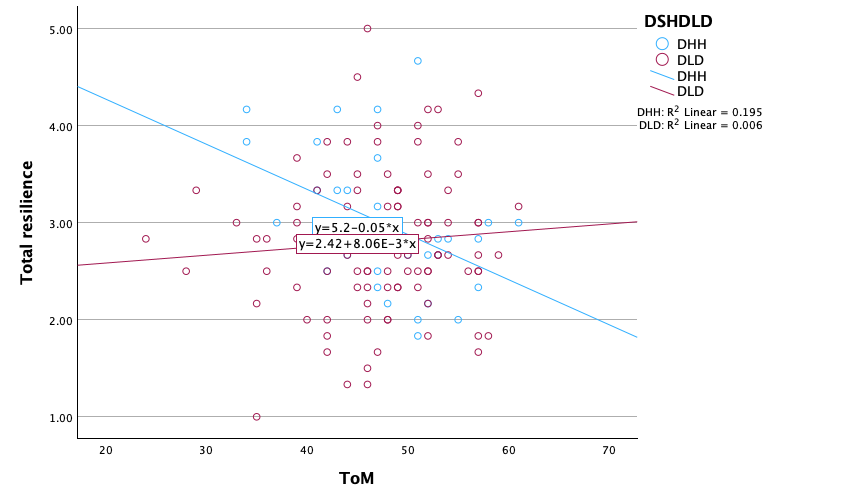
Note: *N* = 127 (DHH *n* = 32, DLD *n* = 95).

**Table 23**

*Percentile Bootstrapping - Interaction of Type of Communication Problems on Regression of ToM, EFs, Schemas, and Social Support to Resilience*

| **Bootstrap Specifications** |  |
| --- | --- |
| Sampling Method | Simple |
| Number of Samples | 1000 |
| Confidence Interval Level | 95.0% |
| Confidence Interval Type | Percentile |

| **Variables Entered/Removed^a^** | Variables Removed | Method |
| --- | --- | --- |
| EFsxTCP, SupxTCP, TCP, ToMCentered, SchemxTCP, EFsCentered, SupportCentered, ToMxTCP, SchemasCentered^b^ | . | Enter |

Note: *N* = 127 (DHH *n* = 32, DLD *n* = 95). TCP = Type of communication problems.

a. Dependent variable: Total resilience. b. All requested variables entered.

| **Model summary** | *R* | *R* square | Adjusted *R* square | Std. error of the estimate |
| --- | --- | --- | --- | --- |
| Model 1 | .623^a^ | .388 | .341 | .60104 |

Note: *N* = 127 (DHH *n* = 32, DLD *n* = 95. a. Predictors: (Constant), TCP, ToM x TCP, Social support, Schemas x TCP, EFs x TCP, EF, Social support x TCP, ToM, Schemas. b. Dependent variable: Total resilience.

| **ANOVA^a^** | Sum of squares | *df* | Mean square | *F* | Sig. |
| --- | --- | --- | --- | --- | --- |
| Regression | 26.813 | 9 | 2.979 | 8.247 | <.001^b^ |
| Residual | 42.267 | 117 | .361 |  |  |
| Total | 69.080 | 126 |  |  |  |

Note: *N* = 127 (DHH *n* = 32, DLD *n* = 95. a. Dependent variable: Total resilience. b. Predictors: (Constant), TCP, ToM x TCP Social support, Schemas x TCP, EFs x TCP, EFs, Social support x TCP, ToM, Schems. **p* < .05.

| **Coefficients^a^** | Unstandardized Coefficients | | Standardized Coefficients | *t* | Sig. |
| --- | --- | --- | --- | --- | --- |
|  | *B* | Std. Error | Beta |  |  |
| (Constant) | 2.891 | .063 |  | 46.035 | <.001 |
| TCP | -.062 | .063 | -.073 | -.984 | .327 |
| SupportCentered | .007 | .018 | .033 | .399 | .691 |
| SchemasCentered | -.599 | .113 | -.528 | -5.319 | <.001 |
| ToMCentered | -.022 | .009 | -.211 | -2.393 | .018 |
| EFsCentered | .004 | .003 | .098 | 1.204 | .231 |
| SchemxTCP | -.026 | .113 | -.023 | -.234 | .815 |
| SupxTCP | .016 | .018 | .073 | .895 | .373 |
| ToMxTCP | .025 | .009 | .241 | 2.729 | .007 |
| EFsxTCP | .003 | .003 | .084 | 1.030 | .305 |

Note: *N* = 127 (DHH *n* = 32, DLD *n* = 95). a. Dependent variable: Total resilience score. Predictors (constant), controlled for type of communication problems (TCP). ToM x TCP, EFs x TCP, Schemas x TCP, Social support x TCP. **p* < .05.

| **Bootstrap for Coefficients** |  | |  | Bootstrap^a^ | 95% Confidence Interval | |
| --- | --- | --- | --- | --- | --- | --- |
| Model 1 | *B* | Bias | Std. Error | Sig. (2-tailed) | Lower | Upper |
| (Constant) | 2.891 | -.007 | .058 | <.001 | 2.768 | 2.995 |
| TCP | -.062 | .008 | .060 | .288 | -.171 | .065 |
| SupportCentered | .007 | .000 | .024 | .752 | -.035 | .059 |
| SchemasCentered | -.599 | .002 | .114 | <.001** | -.828 | -.390 |
| ToMCentered | -.022 | .000 | .009 | .015* | -.040 | -.005 |
| EFsCentered | .004 | .000 | .003 | .204 | -.003 | .010 |
| SchemxTCP | -.026 | -.001 | .111 | .804 | -.250 | .196 |
| SupxTCP | .016 | .001 | .024 | .485 | -.033 | .060 |
| ToMxTCP | .025 | .000 | .008 | .004* | .010 | .042 |
| EFsxTCP | .003 | .000 | .003 | .279 | -.002 | .012 |

Note: *N* = 127 (DHH *n* = 32, DLD *n* = 95). a. Unless otherwise noted, bootstrap results are based on 1000 bootstrap samples. Predictors (constant), controlled for type of communication problems (TCP). ToM x TCP, EFs x TCP, Schemas x TCP, Social support x TCP. **p* < .05.
